# Supplementary figures and images for: PhotoModPlus: A web server for photosynthetic protein prediction from genome neighborhood features
Source: PLoS One. 2021 Mar 17;16(3):e0248682. doi: 10.1371/journal.pone.0248682 (PMC7968678; doi:10.1371/journal.pone.0248682)

**A**

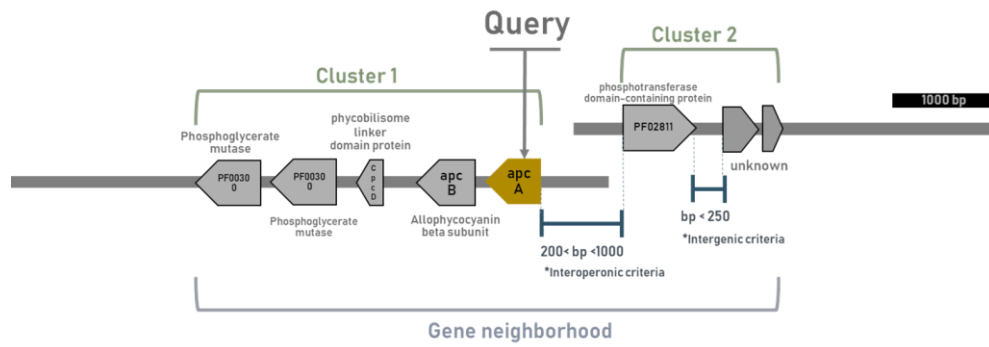

**B**

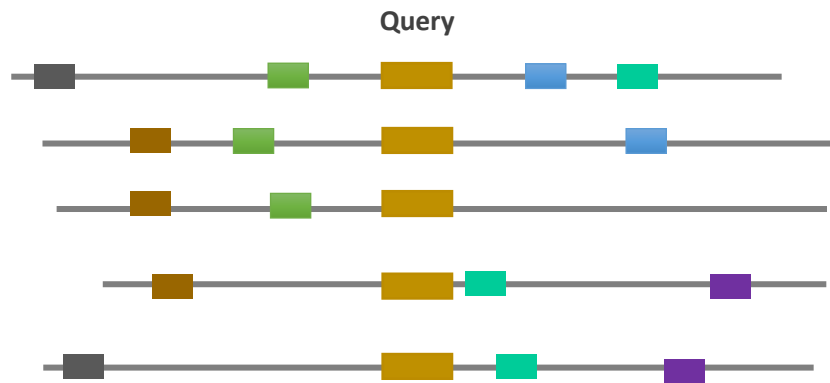

**C**

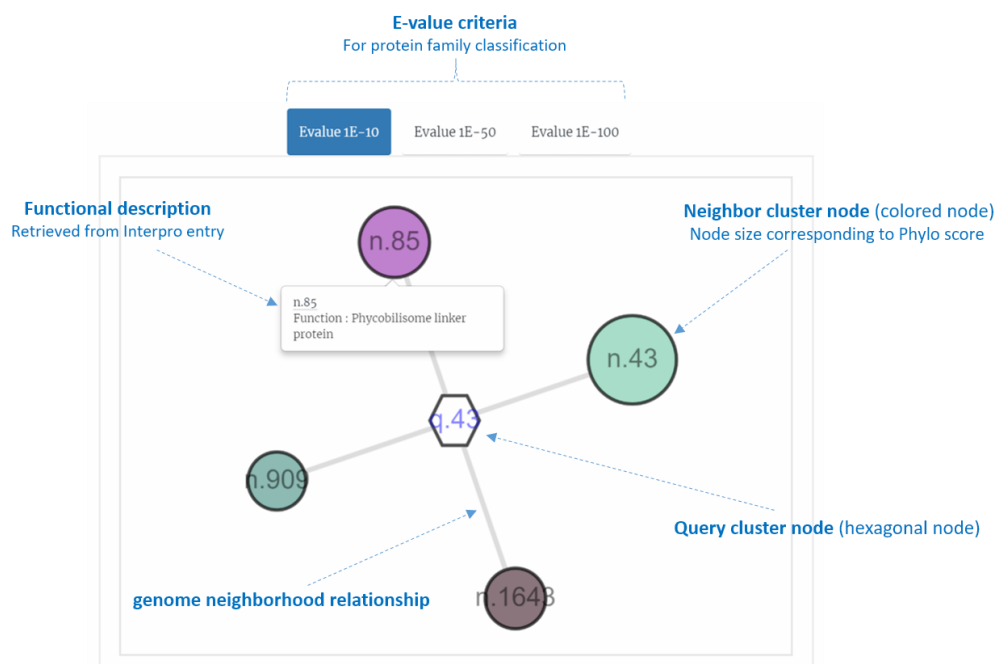

Supplement: S1 Fig — (A) Genes on the same strand are considered neighbors if they are within 250 bp intergenic distance or are overlapping. Additionally, the two clusters are merged into the same neighborhood gene cluster if they are in a range of 200 to 1000 bp in the divergent direction, based on the operon interaction concept [68]. (B) Gene neighborhoods are called from all of the genomes that contain a homolog of the query sequence. (C) After applying protein clustering and calculating the Phylo score, a genome neighborhood network (GNN) can be constructed. The hexagonal node represents the query, while the circular node represents its genome neighbors. The label in each node represents a protein cluster ID. The size of the genome neighbor node varies according to its Phylo score. There are three built-in protein clustering cutoffs (E-value: 1E-10, 1E-50, and 1E-100) available on top of the network to define the level of homologous relationship between proteins. (PDF) [file pone.0248682.s001.pdf]

**A****Photosynthetic dataset**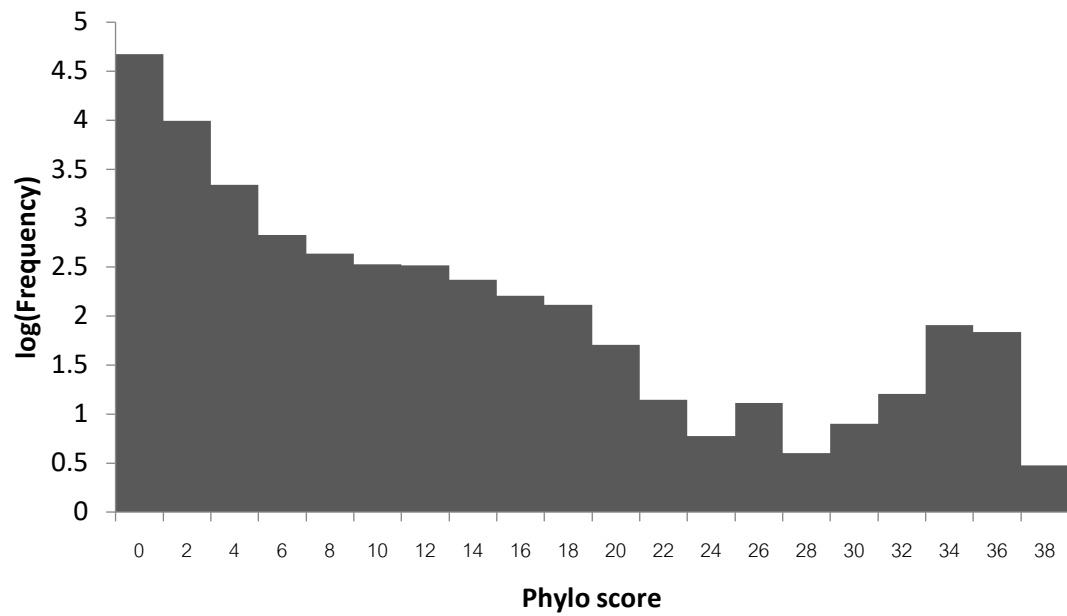**B****Nonphotosynthetic dataset**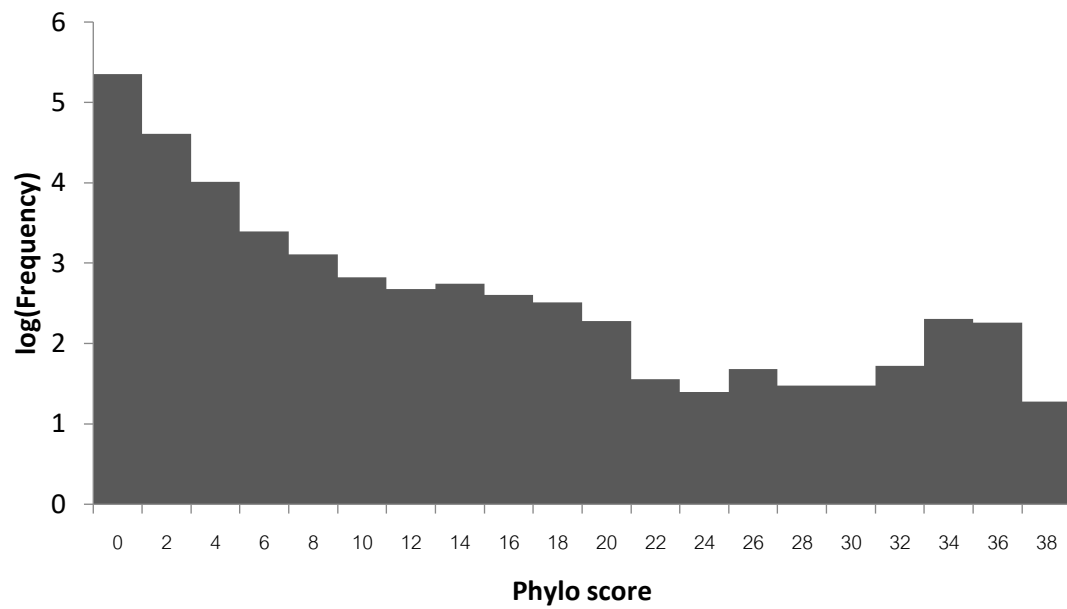

Supplement: S2 Fig — The Phylo scores were collected from the genome neighborhoods of the photosynthetic protein dataset (A) and nonphotosynthetic dataset (B). Note that the zero value of the Phylo score indicates a nonconserved genome neighborhood. (PDF) [file pone.0248682.s002.pdf]

## Outer 5-fold cross-validation

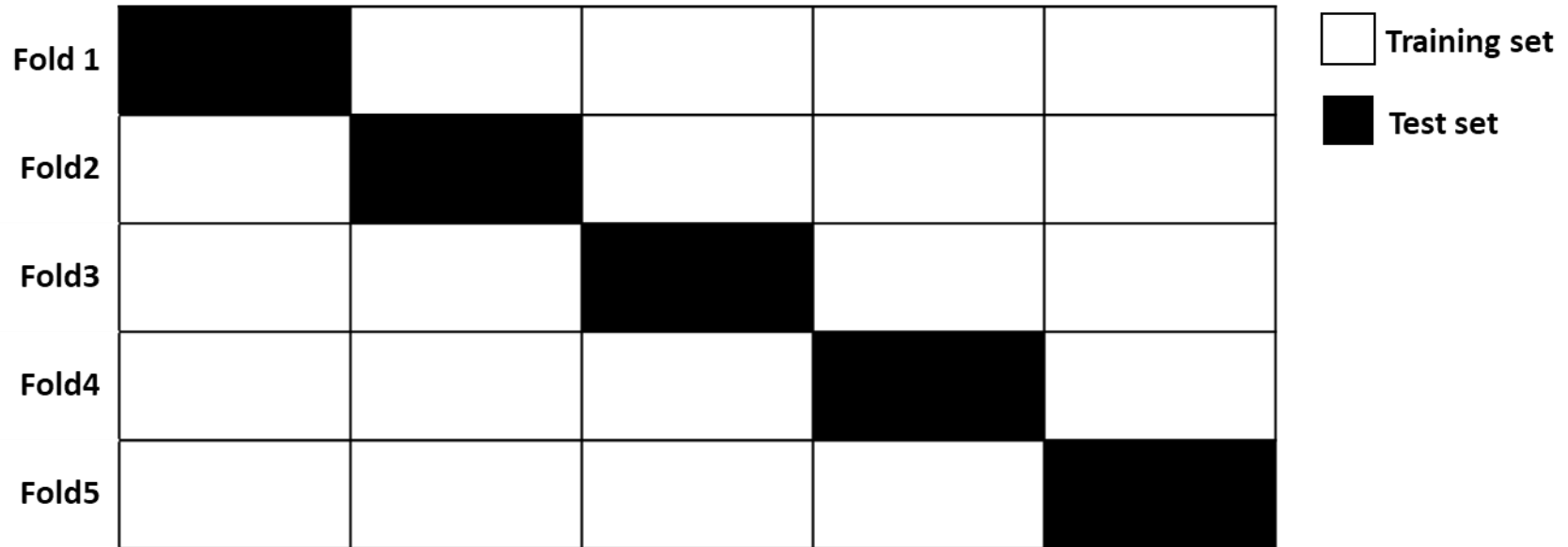

Parameterization

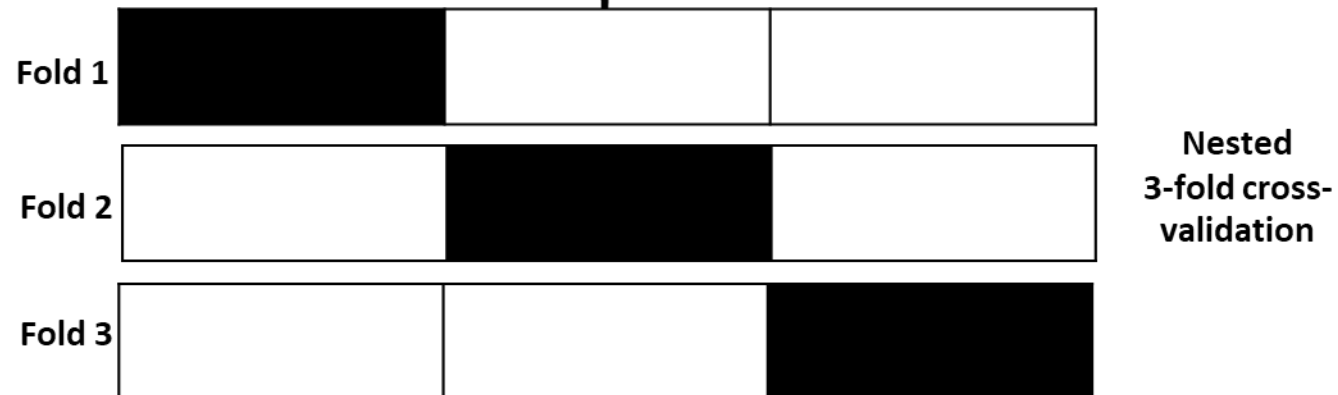

Supplement: S3 Fig — The outer fold is shown in the upper part of the figure, while the nested fold is shown in the lower part. For every outer fold, the training set (white color) is used to perform 3-fold cross-validation to find the best parameter set. The best parameter set is used to build a model again from the whole training set in the outer fold and tested with an independent test set (black color). (PDF) [file pone.0248682.s003.pdf]
