# Supplementary material for: PhotoModPlus: A web server for photosynthetic protein prediction from genome neighborhood features
Source: PLoS One. 2021 Mar 17;16(3):e0248682. doi: 10.1371/journal.pone.0248682 (PMC7968678; doi:10.1371/journal.pone.0248682)
Supplement: S1 Table — (PDF) [file pone.0248682.s004.pdf]

No.	GO term[1]	GO Level (shortest path)	GO Depth (longest path)	Description [main category][2]	N sample	F1		Precision		Recall	
						mean	sd	mean	sd	mean	sd
1	GO:0009521	level-03	depth-04	photosystem [cellular_component]	56	0.699	0.084	0.831	0.204	0.658	0.132
2	GO:0009522	level-04	depth-05	photosystem I [cellular_component]	21	0.740	0.087	0.863	0.141	0.681	0.137
3	GO:0009523	level-04	depth-05	photosystem II [cellular_component]	38	0.545	0.116	0.726	0.262	0.524	0.183
4	GO:0009538	level-03	depth-04	photosystem I reaction center [cellular_component]	8	0.891	0.091	0.905	0.131	0.905	0.131
5	GO:0009539	level-03	depth-04	photosystem II reaction center [cellular_component]	12	0.321	0.105	0.621	0.342	0.390	0.310
6	GO:0009579	level-03	depth-03	thylakoid [cellular_component]	198	0.964	0.022	0.956	0.038	0.973	0.020
7	GO:0009765	level-04	depth-04	photosynthesis, light harvesting [biological_process]	3	0.600	0.490	0.600	0.490	0.600	0.490
8	GO:0009767	level-04	depth-05	photosynthetic electron transport chain [biological_process]	16	0.634	0.172	0.656	0.261	0.731	0.169
9	GO:0009772	level-05	depth-06	photosynthetic electron transport in photosystem II [biological_process]	8	0.467	0.323	0.650	0.436	0.433	0.327
10	GO:0009773	level-05	depth-06	photosynthetic electron transport in photosystem I [biological_process]	2	0.000	0.000	0.000	0.000	0.000	0.000
11	GO:0010109	level-05	depth-05	regulation of photosynthesis [biological_process]	8	0.751	0.198	0.900	0.200	0.657	0.218
12	GO:0010206	level-06	depth-07	photosystem II repair [biological_process]	3	0.400	0.490	0.400	0.490	0.400	0.490
13	GO:0010207	level-06	depth-06	photosystem II assembly [biological_process]	6	0.846	0.158	0.783	0.194	0.933	0.133
14	GO:0019684	level-04	depth-04	photosynthesis, light reaction [biological_process]	46	0.696	0.066	0.728	0.076	0.669	0.065
15	GO:0019685	level-05	depth-05	photosynthesis, dark reaction [biological_process]	28	0.866	0.113	0.853	0.129	0.893	0.137

16	GO:0030089	level-03	depth-04	phycobilisome [cellular_component]	31	0.548	0.243	0.772	0.173	0.489	0.300
17	GO:0030094	level-02	depth-06	plasma membrane-derived photosystem I [cellular_component]	4	0.000	0.000	0.000	0.000	0.000	0.000
18	GO:0030096	level-02	depth-06	plasma membrane-derived thylakoid photosystem II [cellular_component]	15	0.000	0.000	0.000	0.000	0.000	0.000
19	GO:0034357	level-02	depth-04	photosynthetic membrane [cellular_component]	188	0.943	0.032	0.916	0.053	0.974	0.019
20	GO:0042548	level-06	depth-06	regulation of photosynthesis, light reaction [biological_process]	7	0.717	0.163	0.870	0.166	0.657	0.218
21	GO:0042549	level-07	depth-07	photosystem II stabilization [biological_process]	6	0.780	0.221	0.900	0.200	0.707	0.259
22	GO:0043155	level-07	depth-07	negative regulation of photosynthesis, light reaction [biological_process]	2	0.000	0.000	0.000	0.000	0.000	0.000
23	GO:0045156	level-04	depth-04	electron transporter, transferring electrons within the cyclic electron transport pathway of photosynthesis activity [molecular_function]	15	0.397	0.244	0.680	0.412	0.307	0.200
24	GO:0045158	level-04	depth-04	electron transporter, transferring electrons within cytochrome b6/f complex of photosystem II activity [molecular_function]	8	0.700	0.367	0.800	0.400	0.640	0.367

[1]All child terms of photosynthesis (GO:0015979) and all of their child terms connected by “is_a” and “part_of” relationship were extracted recursively. This generates large list of photosynthetic related GO terms located in all three main categories (Molecular Function, Biological Process, and Cellular Component). The GO terms that also participate in other function in Biological Process category were removed. The remaining terms were considered as photosynthetic specific GO terms. In addition, we manually added phycobilisome term (GO:0030089). Although this GO term is not the child term of photosynthesis, it can be considered as marginally connected term to photosynthesis through photosynthetic membrane (GO:0034357).

[2]Gene ontology file (go-basic.obo) version 1.2 updated on 2019-07-01 47,413 GO terms
