# Supplementary material for: PhotoModPlus: A web server for photosynthetic protein prediction from genome neighborhood features
Source: PLoS One. 2021 Mar 17;16(3):e0248682. doi: 10.1371/journal.pone.0248682 (PMC7968678; doi:10.1371/journal.pone.0248682)
Supplement: S2 Table — (PDF) [file pone.0248682.s005.pdf]

	PhotoModGO-RAkEL	PhotoModGO-BR	PhotoModGO-LP	DeepGOPlus	BLAST
PhotoModGO-RAkEL	-	-	-	-	-
PhotoModGO-BR	0.166	-	-	-	-
PhotoModGO-LP	0.000	0.000	-	-	-
DeepGOPlus	0.000	0.000	0.135	-	-
BLAST	0.000	0.000	0.000	0.000	-
