# Supplementary material for: PhotoModPlus: A web server for photosynthetic protein prediction from genome neighborhood features
Source: PLoS One. 2021 Mar 17;16(3):e0248682. doi: 10.1371/journal.pone.0248682 (PMC7968678; doi:10.1371/journal.pone.0248682)
Supplement: S3 Table — (PDF) [file pone.0248682.s006.pdf]

Method	Gene	Predicted GO	Probability	Description
DeepGOplus	rfpA	GO:0034357	0.405	photosynthetic membrane
		GO:0009579	0.434	thylakoid
	rfpB	GO:0034357	0.396	photosynthetic membrane
		GO:0009579	0.426	thylakoid
	IflA	GO:0034357	0.410	photosynthetic membrane
		GO:0009579	0.435	thylakoid
	DpxA	GO:0034357	0.398	photosynthetic membrane
		GO:0009579	0.429	thylakoid
	fciA	GO:0034357	0.388	photosynthetic membrane
		GO:0009579	0.429	thylakoid
	fciB	GO:0034357	0.389	photosynthetic membrane
		GO:0009579	0.425	thylakoid
	isiX	GO:0034357	0.432	photosynthetic membrane
		GO:0019684	0.189	photosynthesis
		GO:0009579	0.451	thylakoid
	apcD4	GO:0034357	0.459	photosynthetic membrane
		GO:0009579	0.474	thylakoid
	apcB3	GO:0034357	0.471	photosynthetic membrane
		GO:0009579	0.482	thylakoid
		GO:0030089	0.163	phycobilisome
	MpeZ	GO:0034357	0.395	photosynthetic membrane
		GO:0009579	0.423	thylakoid
	slr0151	GO:0034357	0.384	photosynthetic membrane
		GO:0009579	0.424	thylakoid
	CyanoP	GO:0034357	0.446	photosynthetic membrane
		GO:0009579	0.466	thylakoid
	Slr1658	GO:0034357	0.432	photosynthetic membrane
		GO:0009579	0.452	thylakoid
	sll0272	GO:0034357	0.446	photosynthetic membrane
		GO:0009521	0.141	Photosystem
		GO:0009579	0.465	thylakoid
	ssl3829	GO:0034357	0.414	photosynthetic membrane
		GO:0009523	0.122	photosystem II
		GO:0009521	0.177	Photosystem
		GO:0019684	0.104	photosynthesis
		GO:0009579	0.446	thylakoid
	asr1131	GO:0034357	0.420	photosynthetic membrane
		GO:0009523	0.149	photosystem II
		GO:0009521	0.210	Photosystem
		GO:0019684	0.123	photosynthesis
		GO:0009579	0.450	thylakoid
	slr1188	GO:0034357	0.417	photosynthetic membrane
		GO:0009579	0.440	thylakoid
	all4940	GO:0034357	0.446	photosynthetic membrane
		GO:0009579	0.469	thylakoid
SVMprot	rfpB	GO:0009522	0.586	photosystem I
	fciA	GO:0009522	0.586	photosystem I
	isiX	GO:0009523	0.854	photosystem II
	MpeZ	GO:0009522	0.586	photosystem I
	CyanoP	GO:0009522	0.586	photosystem I
	all4940	GO:0009522	0.586	photosystem I
