# Supplementary material for: PhotoModPlus: A web server for photosynthetic protein prediction from genome neighborhood features
Source: PLoS One. 2021 Mar 17;16(3):e0248682. doi: 10.1371/journal.pone.0248682 (PMC7968678; doi:10.1371/journal.pone.0248682)
Supplement: S4 Table — The unknown protein-coding genes from Synechocystissp. PCC 6803 genome (1,885 genes) were used as input in PhotoModPlus prediction. BLAST best match and E-value of 1E-25 were used as BLAST parameters. The potential candidates were selected using the criteria: i) predicted as a positive class with more than 80% probability from the PhotoMod binary model and ii) containing at least one predicted GO term with more than 50% probability from the PhotoModGO multi-label model. (PDF) [file pone.0248682.s007.pdf]

Locus	Predicted GO term	Pro babi lity	Description
sll0071 hypothetical protein	GO:0009579	1.0	thylakoid
sll0088 hypothetical protein	GO:0009579	1.0	thylakoid
sll0088 hypothetical protein	GO:0034357	1.0	photosynthetic membrane
sll0160 hypothetical protein	GO:0009579	1.0	thylakoid
sll0185 hypothetical protein	GO:0009579	1.0	thylakoid
sll0185 hypothetical protein	GO:0034357	1.0	photosynthetic membrane
sll0249 hypothetical protein	GO:0009521	1.0	Photosystem
sll0249 hypothetical protein	GO:0009522	1.0	photosystem I
sll0249 hypothetical protein	GO:0009579	1.0	thylakoid
sll0249 hypothetical protein	GO:0034357	1.0	photosynthetic membrane
sll0272 hypothetical protein	GO:0009521	1.0	Photosystem
sll0272 hypothetical protein	GO:0009523	1.0	photosystem II
sll0272 hypothetical protein	GO:0009579	1.0	thylakoid
sll0272 hypothetical protein	GO:0010109	1.0	regulation of photosynthesis
sll0272 hypothetical protein	GO:0019684	1.0	photosynthesis, light reactions
sll0272 hypothetical protein	GO:0034357	1.0	photosynthetic membrane
sll0272 hypothetical protein	GO:0042548	1.0	regulation of photosynthesis, light reaction
sll0272 hypothetical protein	GO:0042549	1.0	photosystem II stabilization
sll0364 hypothetical protein	GO:0009579	1.0	thylakoid
sll0436 hypothetical protein	GO:0019684	1.0	photosynthesis, light reactions
sll0436 hypothetical protein	GO:0045156	0.6	electron transporter, transferring electrons within the cyclic electron transport pathway of photosynthesis
sll0611 hypothetical protein	GO:0009521	1.0	Photosystem
sll0611 hypothetical protein	GO:0009523	1.0	photosystem II
sll0611 hypothetical protein	GO:0009539	1.0	photosystem II reaction center
sll0611 hypothetical protein	GO:0009579	1.0	thylakoid
sll0611 hypothetical protein	GO:0030096	0.9	photosystem II (sensu Cyanobacteria)
sll0611 hypothetical protein	GO:0034357	1.0	photosynthetic membrane
sll1285 hypothetical protein	GO:0009579	1.0	thylakoid
sll1340 hypothetical protein	GO:0009579	1.0	thylakoid
sll1340 hypothetical protein	GO:0034357	1.0	photosynthetic membrane
sll1399 hypothetical protein	GO:0009579	1.0	thylakoid
sll1399 hypothetical protein	GO:0034357	1.0	photosynthetic membrane
sll1400 hypothetical protein	GO:0009521	0.6	Photosystem
sll1400 hypothetical protein	GO:0009579	1.0	thylakoid
sll1400 hypothetical protein	GO:0034357	1.0	photosynthetic membrane
sll1414 hypothetical protein	GO:0010207	1.0	photosystem II assembly
sll1414 hypothetical protein	GO:0019684	1.0	photosynthesis, light reactions
sll1573 hypothetical protein	GO:0009579	0.8	thylakoid
sll1757 hypothetical protein	GO:0009521	0.6	Photosystem
sll1757 hypothetical protein	GO:0009579	1.0	thylakoid
sll1757 hypothetical protein	GO:0034357	1.0	photosynthetic membrane
sll1874 hypothetical protein	GO:0019685	1.0	photosynthesis, dark reactions
sll1916 hypothetical protein	GO:0009579	1.0	thylakoid
sll1979 hypothetical protein	GO:0009579	1.0	thylakoid
sll1979 hypothetical protein	GO:0034357	1.0	photosynthetic membrane
sll7034 hypothetical protein	GO:0009579	1.0	thylakoid
sll7034 hypothetical protein	GO:0034357	1.0	photosynthetic membrane
slr0022 hypothetical protein	GO:0009521	1.0	Photosystem
slr0022 hypothetical protein	GO:0009523	1.0	photosystem II
slr0022 hypothetical protein	GO:0009579	1.0	thylakoid
slr0022 hypothetical protein	GO:0010109	1.0	regulation of photosynthesis
slr0022 hypothetical protein	GO:0019684	1.0	photosynthesis, light reactions
slr0022 hypothetical protein	GO:0034357	1.0	photosynthetic membrane

slr0022 hypothetical protein	GO:0042548	1.0	regulation of photosynthesis, light reaction
slr0022 hypothetical protein	GO:0042549	1.0	photosystem II stabilization
slr0076 hypothetical protein	GO:0009579	1.0	thylakoid
slr0076 hypothetical protein	GO:0034357	1.0	photosynthetic membrane
slr0148 hypothetical protein	GO:0009579	0.8	thylakoid
slr0249 hypothetical protein	GO:0009579	1.0	thylakoid
slr0249 hypothetical protein	GO:0034357	1.0	photosynthetic membrane
slr0250 hypothetical protein	GO:0009579	1.0	thylakoid
slr0250 hypothetical protein	GO:0034357	1.0	photosynthetic membrane
slr0388 hypothetical protein	GO:0009521	1.0	Photosystem
slr0388 hypothetical protein	GO:0009523	1.0	photosystem II
slr0388 hypothetical protein	GO:0009579	1.0	thylakoid
slr0388 hypothetical protein	GO:0034357	1.0	photosynthetic membrane
slr0598 hypothetical protein	GO:0009579	1.0	thylakoid
slr0770 hypothetical protein	GO:0009579	1.0	thylakoid
slr0770 hypothetical protein	GO:0034357	1.0	photosynthetic membrane
slr0771 hypothetical protein	GO:0009521	0.6	Photosystem
slr0771 hypothetical protein	GO:0009579	1.0	thylakoid
slr0771 hypothetical protein	GO:0034357	1.0	photosynthetic membrane
slr0869 hypothetical protein	GO:0009579	1.0	thylakoid
slr1100 hypothetical protein	GO:0009579	1.0	thylakoid
slr1100 hypothetical protein	GO:0034357	1.0	photosynthetic membrane
slr1170 hypothetical protein	GO:0009579	1.0	thylakoid
slr1170 hypothetical protein	GO:0034357	1.0	photosynthetic membrane
slr1183 hypothetical protein	GO:0009521	0.6	Photosystem
slr1183 hypothetical protein	GO:0009579	1.0	thylakoid
slr1183 hypothetical protein	GO:0034357	1.0	photosynthetic membrane
slr1186 hypothetical protein	GO:0009579	1.0	thylakoid
slr1186 hypothetical protein	GO:0034357	1.0	photosynthetic membrane
slr1195 hypothetical protein	GO:0009579	1.0	thylakoid
slr1209 hypothetical protein	GO:0009579	1.0	thylakoid
slr1209 hypothetical protein	GO:0034357	1.0	photosynthetic membrane
slr1220 hypothetical protein	GO:0009521	0.6	Photosystem
slr1220 hypothetical protein	GO:0009579	1.0	thylakoid
slr1220 hypothetical protein	GO:0034357	1.0	photosynthetic membrane
slr1394 hypothetical protein	GO:0009579	1.0	thylakoid
slr1394 hypothetical protein	GO:0034357	1.0	photosynthetic membrane
slr1495 hypothetical protein	GO:0009579	1.0	thylakoid
slr1495 hypothetical protein	GO:0034357	1.0	photosynthetic membrane
slr1601 hypothetical protein	GO:0009521	0.6	Photosystem
slr1601 hypothetical protein	GO:0009579	1.0	thylakoid
slr1601 hypothetical protein	GO:0034357	1.0	photosynthetic membrane
slr1699 hypothetical protein	GO:0009579	1.0	thylakoid
slr1699 hypothetical protein	GO:0034357	1.0	photosynthetic membrane
slr1732 hypothetical protein	GO:0009579	0.8	thylakoid
slr1767 hypothetical protein	GO:0009521	0.6	Photosystem
slr1767 hypothetical protein	GO:0009579	1.0	thylakoid
slr1767 hypothetical protein	GO:0034357	1.0	photosynthetic membrane
slr1927 hypothetical protein	GO:0009521	0.6	Photosystem
slr1927 hypothetical protein	GO:0009579	1.0	thylakoid
slr1927 hypothetical protein	GO:0034357	1.0	photosynthetic membrane
slr1998 hypothetical protein	GO:0009521	0.8	Photosystem
slr1998 hypothetical protein	GO:0009523	1.0	photosystem II
slr1998 hypothetical protein	GO:0009579	1.0	thylakoid
slr1998 hypothetical protein	GO:0034357	0.8	photosynthetic membrane
slr2070 hypothetical protein	GO:0009521	0.6	Photosystem
slr2070 hypothetical protein	GO:0009579	1.0	thylakoid
slr2070 hypothetical protein	GO:0034357	1.0	photosynthetic membrane

ssl0385 hypothetical protein	GO:0009579	1.0	thylakoid
ssl0385 hypothetical protein	GO:0034357	1.0	photosynthetic membrane
ssl0483 hypothetical protein	GO:0009521	1.0	Photosystem
ssl0483 hypothetical protein	GO:0009523	1.0	photosystem II
ssl0483 hypothetical protein	GO:0009579	1.0	thylakoid
ssl0483 hypothetical protein	GO:0019684	1.0	photosynthesis, light reactions
ssl0483 hypothetical protein	GO:0034357	1.0	photosynthetic membrane
ssl2717 hypothetical protein	GO:0009579	1.0	thylakoid
ssl2717 hypothetical protein	GO:0034357	1.0	photosynthetic membrane
ssl2920 hypothetical protein	GO:0009521	0.6	Photosystem
ssl2920 hypothetical protein	GO:0009579	1.0	thylakoid
ssl2920 hypothetical protein	GO:0034357	1.0	photosynthetic membrane
ssl2921 hypothetical protein	GO:0009521	0.6	Photosystem
ssl2921 hypothetical protein	GO:0009579	1.0	thylakoid
ssl2921 hypothetical protein	GO:0034357	1.0	photosynthetic membrane
ssr1558 hypothetical protein	GO:0009579	1.0	thylakoid
ssr1558 hypothetical protein	GO:0034357	1.0	photosynthetic membrane
ssr1698 hypothetical protein	GO:0009521	1.0	Photosystem
ssr1698 hypothetical protein	GO:0009523	1.0	photosystem II
ssr1698 hypothetical protein	GO:0009579	1.0	thylakoid
ssr1698 hypothetical protein	GO:0034357	1.0	photosynthetic membrane
ssr2781 hypothetical protein	GO:0009579	1.0	thylakoid
ssr2781 hypothetical protein	GO:0034357	1.0	photosynthetic membrane
ssr5011 hypothetical protein	GO:0009579	1.0	thylakoid
sll0047 hypothetical protein YCF12	GO:0009521	0.6	Photosystem
sll0047 hypothetical protein YCF12	GO:0009579	1.0	thylakoid
sll0047 hypothetical protein YCF12	GO:0034357	1.0	photosynthetic membrane
sll1509 hypothetical protein YCF20	GO:0009579	1.0	thylakoid
slr2049 hypothetical protein YCF58	GO:0009579	1.0	thylakoid
slr2049 hypothetical protein YCF58	GO:0030089	1.0	phycobilisome
slr2049 hypothetical protein YCF58	GO:0034357	1.0	photosynthetic membrane
sll1214 hypothetical protein YCF59	GO:0019685	1.0	photosynthesis, dark reactions
sll0048 unknown protein	GO:0009521	0.6	Photosystem
sll0048 unknown protein	GO:0009579	1.0	thylakoid
sll0048 unknown protein	GO:0034357	1.0	photosynthetic membrane
sll0225 unknown protein	GO:0009579	1.0	thylakoid
sll0225 unknown protein	GO:0034357	1.0	photosynthetic membrane
sll0263 unknown protein	GO:0009579	1.0	thylakoid
sll0263 unknown protein	GO:0034357	1.0	photosynthetic membrane
sll0265 unknown protein	GO:0009579	1.0	thylakoid
sll0265 unknown protein	GO:0034357	1.0	photosynthetic membrane
sll0266 unknown protein	GO:0009579	1.0	thylakoid
sll0266 unknown protein	GO:0034357	1.0	photosynthetic membrane
sll0293 unknown protein	GO:0009521	0.6	Photosystem
sll0293 unknown protein	GO:0009579	1.0	thylakoid
sll0293 unknown protein	GO:0034357	1.0	photosynthetic membrane
sll0327 unknown protein	GO:0009521	0.6	Photosystem
sll0327 unknown protein	GO:0009579	1.0	thylakoid
sll0327 unknown protein	GO:0034357	1.0	photosynthetic membrane
sll0328 unknown protein	GO:0009521	0.6	Photosystem

sll0328 unknown protein	GO:0009579	1.0	thylakoid
sll0328 unknown protein	GO:0034357	1.0	photosynthetic membrane
sll0539 unknown protein	GO:0009521	0.6	Photosystem
sll0539 unknown protein	GO:0009579	1.0	thylakoid
sll0539 unknown protein	GO:0034357	1.0	photosynthetic membrane
sll0552 unknown protein	GO:0009579	1.0	thylakoid
sll0552 unknown protein	GO:0034357	1.0	photosynthetic membrane
sll0623 unknown protein	GO:0009521	0.6	Photosystem
sll0623 unknown protein	GO:0009579	1.0	thylakoid
sll0623 unknown protein	GO:0034357	1.0	photosynthetic membrane
sll1268 unknown protein	GO:0009521	0.6	Photosystem
sll1268 unknown protein	GO:0009579	1.0	thylakoid
sll1268 unknown protein	GO:0034357	1.0	photosynthetic membrane
sll1401 unknown protein	GO:0009521	0.6	Photosystem
sll1401 unknown protein	GO:0009579	1.0	thylakoid
sll1401 unknown protein	GO:0034357	1.0	photosynthetic membrane
sll1503 unknown protein	GO:0009521	0.6	Photosystem
sll1503 unknown protein	GO:0009579	1.0	thylakoid
sll1503 unknown protein	GO:0034357	1.0	photosynthetic membrane
sll1510 unknown protein	GO:0009521	0.6	Photosystem
sll1510 unknown protein	GO:0009579	1.0	thylakoid
sll1510 unknown protein	GO:0034357	1.0	photosynthetic membrane
sll1511 unknown protein	GO:0009521	0.6	Photosystem
sll1511 unknown protein	GO:0009579	1.0	thylakoid
sll1511 unknown protein	GO:0034357	1.0	photosynthetic membrane
sll1763 unknown protein	GO:0009521	0.6	Photosystem
sll1763 unknown protein	GO:0009579	1.0	thylakoid
sll1763 unknown protein	GO:0034357	1.0	photosynthetic membrane
sll1764 unknown protein	GO:0009521	0.6	Photosystem
sll1764 unknown protein	GO:0009579	1.0	thylakoid
sll1764 unknown protein	GO:0034357	1.0	photosynthetic membrane
sll1765 unknown protein	GO:0009521	0.6	Photosystem
sll1765 unknown protein	GO:0009579	1.0	thylakoid
sll1765 unknown protein	GO:0034357	1.0	photosynthetic membrane
sll1830 unknown protein	GO:0009521	0.6	Photosystem
sll1830 unknown protein	GO:0009579	1.0	thylakoid
sll1830 unknown protein	GO:0034357	1.0	photosynthetic membrane
slr0059 unknown protein	GO:0009521	0.6	Photosystem
slr0059 unknown protein	GO:0009579	1.0	thylakoid
slr0059 unknown protein	GO:0034357	1.0	photosynthetic membrane
slr0145 unknown protein	GO:0009579	1.0	thylakoid
slr0151 unknown protein	GO:0009579	1.0	thylakoid
slr0196 unknown protein	GO:0009521	0.6	Photosystem
slr0196 unknown protein	GO:0009579	1.0	thylakoid
slr0196 unknown protein	GO:0034357	1.0	photosynthetic membrane
slr0226 unknown protein	GO:0009521	0.6	Photosystem
slr0226 unknown protein	GO:0009579	1.0	thylakoid
slr0226 unknown protein	GO:0034357	1.0	photosynthetic membrane
slr0421 unknown protein	GO:0009579	1.0	thylakoid
slr0442 unknown protein	GO:0009521	0.6	Photosystem
slr0442 unknown protein	GO:0009579	1.0	thylakoid
slr0442 unknown protein	GO:0034357	1.0	photosynthetic membrane
slr0602 unknown protein	GO:0009579	1.0	thylakoid
slr0602 unknown protein	GO:0034357	1.0	photosynthetic membrane
slr1168 unknown protein	GO:0009579	1.0	thylakoid
slr1168 unknown protein	GO:0034357	1.0	photosynthetic membrane
slr1187 unknown protein	GO:0009521	0.6	Photosystem
slr1187 unknown protein	GO:0009579	1.0	thylakoid

slr1187 unknown protein	GO:0034357	1.0	photosynthetic membrane
slr1189 unknown protein	GO:0009521	0.6	Photosystem
slr1189 unknown protein	GO:0009579	1.0	thylakoid
slr1189 unknown protein	GO:0034357	1.0	photosynthetic membrane
slr1567 unknown protein	GO:0009521	0.6	Photosystem
slr1567 unknown protein	GO:0009579	1.0	thylakoid
slr1567 unknown protein	GO:0034357	1.0	photosynthetic membrane
slr1576 unknown protein	GO:0009521	0.6	Photosystem
slr1576 unknown protein	GO:0009579	1.0	thylakoid
slr1576 unknown protein	GO:0034357	1.0	photosynthetic membrane
ssl0738 unknown protein	GO:0009521	0.6	Photosystem
ssl0738 unknown protein	GO:0009579	1.0	thylakoid
ssl0738 unknown protein	GO:0034357	1.0	photosynthetic membrane
ssl2138 unknown protein	GO:0009579	1.0	thylakoid
ssl2138 unknown protein	GO:0034357	1.0	photosynthetic membrane
ssl3142 unknown protein	GO:0009521	0.6	Photosystem
ssl3142 unknown protein	GO:0009579	1.0	thylakoid
ssl3142 unknown protein	GO:0034357	1.0	photosynthetic membrane
